# Trehalose and Trehalose-based Polymers for Environmentally Benign, Biocompatible and Bioactive Materials

**DOI:** 10.3390/molecules13081773

**Published:** 2008-08-21

**Authors:** Naozumi Teramoto, Navzer D. Sachinvala, Mitsuhiro Shibata

**Affiliations:** 1 Department of Life and Environmental Sciences, Faculty of Engineering, Chiba Institute of Technology, 2-17-1 Tsudanuma, Narashino, Chiba 275-0016, Japan; E-mail: teramoto-n@sea.it-chiba.ac.jp; 2 Retired, Southern Regional Research Center, USDA-ARS, New Orleans, LA, USA; Home: 2261 Brighton Place, Harvey, LA 70058; E-mail: Sachinvala@cox.net

**Keywords:** Trehalose, Cryptobiosis, Biopreservation, Trehalose-based monomers, Linear polymers, Network polymers, Biocompatible polymers

## Abstract

Trehalose is a non-reducing disaccharide that is found in many organisms but not in mammals. This sugar plays important roles in cryptobiosis of selaginella mosses, tardigrades (water bears), and other animals which revive with water from a state of suspended animation induced by desiccation. The interesting properties of trehalose are due to its unique symmetrical low-energy structure, wherein two glucose units are bonded face-to-face by 1→1-glucoside links. The Hayashibara Co. Ltd., is credited for developing an inexpensive, environmentally benign and industrial-scale process for the enzymatic conversion of α-1,4-linked polyhexoses to α,α-d-trehalose, which made it easy to explore novel food, industrial, and medicinal uses for trehalose and its derivatives. Trehalose-chemistry is a relatively new and emerging field, and polymers of trehalose derivatives appear environmentally benign, biocompatible, and biodegradable. The discriminating properties of trehalose are attributed to its structure, symmetry, solubility, kinetic and thermodynamic stability and versatility. While syntheses of trehalose-based polymer networks can be straightforward, syntheses and characterization of well defined linear polymers with tailored properties using trehalose-based monomers is challenging, and typically involves protection and deprotection of hydroxyl groups to attain desired structural, morphological, biological, and physical and chemical properties in the resulting products. In this review, we will overview known literature on trehalose’s fascinating involvement in cryptobiology; highlight its applications in many fields; and then discuss methods we used to prepare new trehalose-based monomers and polymers and explain their properties.

## Introduction

Trehalose is a non-reducing disaccharide found in bacteria, fungi, plants and invertebrates such as insects and nematodes. However, its existence and biosynthesis in mammals are not known [1]. The sugar was named by Berthelot in 1858 who found it in *trehala*, a desert manna produced by the weevil *Larinus nidificans* [2]. Prior to Berthelot, Wiggers discovered trehalose in 1832 while studying solutions of ergot of rye, a plant disease caused by the fungus *Claviceps purpurea* [3]. Winged insects produce trehalose as an energy source in their haemolymph (blood-lymph system) to generate blood glucose in high concentrations as a source of energy for flight [4, 5, 6]. The concentration of trehalose in insect haemolymph is usually between 1% and 2% [7], whereas the concentration of glucose in humans is about 0.1%. The disaccharide is a novel food chemical, generally regarded as safe (GRAS), and can be stored in relatively high concentrations in body fluids. This is because trehalose is non-reducing, unlike glucose (a reducing sugar), which at higher serum concentrations is toxic to humans.

Trehalase (α,α-trehalose-1-C-glucohydrolase, EC 3.2.1.28), the corresponding enzyme that specifically cleaves trehalose into two molecules of glucose, was first reported in extracts of *Aspergilus niger* [8]. The enzyme is not ubiquitous in mammal bodies and therefore serves as a highly sensitive analytical tool to detect trehalose in biological samples [3, 9, 10]. Nonetheless, dietary trehalose is rapidly cleaved as the glycoprotein (trehalase, enzyme) is found in the intestinal villi of mammals [3, 11]. It is found also in brush border membranes of proximal renal tubules [3, 12]; however, the presence of trehalase in the urine of patients usually signifies nephritis or acute phase anaphylaxis [13]. The gene for trehalase in humans was isolated, cloned, and enzyme-linked immunosorbent assays (ELISA) for its clinical uses were developed [14, 15]. In addition, individuals with a defect in intestinal trehalase have diarrhea and discomfort following consumption of foods high in trehalose content [16, 17]. Consequently, much regarding the role of trehalase in humans awaits discovery. 

Trehalose is present also in yeast and fungi; in spores, fruiting bodies, and during the induction of growth in vegetative (resting) cells [18, 19, 20, 21, 22, 23]. There exists 7% (on the basis of dry weight) trehalose in the spores and macrocysts of *Dictyostelium mucoroides*, and 10% trehalose in the ascospores of *Neurospora tetrasperma*. When these spores germinate, the concentration of trehalose decreased rapidly, and it is likely that trehalose was stored as a source of energy for growth. 

More recently, trehalose was reported to protect the integrity of cells in many organisms against environmental stresses due to its physical, colligative, and chemical properties [24, 25]. Cryptobiosis is a state in organisms when no visible signs of life appear, and animals become ametabolic. That is, the activity either becomes hardly measurable, or reversibly ceases [26, 27, 28]. Terms used for cryptobiosis (means suspended animation and revival) for organisms experiencing various forms of environmental stresses are: anhydrobiosis (desiccation), cryobiosis (low temperature), anoxybiosis (lack of oxygen), and osmobiosis (high pressure), respectively. 

In 1702 the Dutch microscopist Antoine Van Leeuwenhoek discovered the phenomenon of cryptobiosis in organisms found in his roof gutter dust. When viewing the dry dust under a microscope, the powder appeared lifeless. However, when the powder was treated with clean water in a glass tube, many small 'animalcules' became active within an hour; some ‘adhering to the glass, some creeping along the walls of the tube, and some swimming about’ [29]. Furthermore, the phenomenon was reproducible, even when the roof dust was kept dry for several months. Historically, Leeuwenhoek’s discovery is the first report on reviving desiccated bdelloid rotifers and cryptobiosis. Rotifers are microscopic animals found in fresh water. They are asexual, metazoic, multicellular, and parthenogenetic organisms with a rotating head and many cilia. Bdelloid (which means "leach like") rotifers have an elongated body, a brain, two eyes, alimentary and nervous systems, cilia mediated sensing, and a pseudopodium (foot-like organ) for motility. 

Other documented examples of prolonged anhydrobiosis include the eggs of brine shrimp, *Artemia*, which were maintained for 15 years [30], survival of rotifers up to 9 years [31] and tardigrades (water bears) that survived following water removal for just as long [32, 33, 34]. 

Tardigrades also show extraordinary tolerance for other stresses, *viz*; high-energy radiation, immersion in organic solvents, brief incubation in excess of 100°C, and survival in the Himalayas at +6,000 m, and oceans at -4,000 m. The animals uniquely have thick cylindrical bodies consisting of four segments, four pairs of legs without joints, feet with claws or toes, and ventral nervous system. In recent experiments on lichen-dwelling tardigrades anhydrobiosis was observed by Rebecchi *et al*. [35]. The first significant recovery of *Ramazzottius oberhaeuseri* was observed after 86 days under atmospheric oxygen at ambient temperature; and the longest term for survival of 1604 days was also recorded in the experiment. Ramløv and Westh froze tardigrades, *Adorybiotus coronifer*, to -196°C at various rates up to 1500°C min^-1^ and found that cryobiosis proceeded concomitantly with anhydrobiosis [33, 36]. They found that viability of animals decreased with faster cooling rates, while several animals (ca. 20%) survived cooling at 400°C per min. This suggested that trehalose and other solutes in the milieu during cooling influenced the formation of amorphous structures (instead of ice crystals with sharp edges) which protected the animal from suffering tissue injury (explained below). Furthermore, once animals are cooled below ca. -7°C at a lower cooling rates, subsequent shock-cooling has little or no effect on their viability. During cryptobiosis, metabolic activities could not be detectable, but the degree of the depression of metabolism remains poorly understood. Due to its reversibility, cryptobiosis is a unique biological state of suspended animation between life and death, and roles of disaccharides, polyhydroxy compounds, and proteins therein deserve much investigation. 

Researchers have alluded to the contribution of non-reducing disaccharide, especially trehalose in anhydrobiotic animals, fungi some resurrection plants such as selaginella, and bacteria. Clegg and colleagues studied the involvement of high concentrations of polyhydroxy compounds and first revealed that trehalose at concentrations of about 15% (of dry weight) in *Artemia* cysts played the important role in the organism's ability to tolerate desiccation [20, 30, 37, 38, 39, 40]. 

Crowe showed the strong correlation between trehalose concentration and desiccation tolerance of nematodes, *Aphelenchus avenae* wherein trehalose was found to be as much as 20% dry weight (of nematode). He also found that trehalose concentrations increased in a tardigrade, *Macrobiotus areolatus*, during anhydrobiosis, but accumulated trehalose was only up to 2% dry weight and was lower than expected [29, 33, 41, 42, 43, 44].

Crowe coined the "water replacement" hypothesis to explain the role of polyhydroxy compounds including trehalose in cryptobiosis [45, 46, explained in detail below]. The first quantitative study on the accumulation of trehalose and polyhydroxy compounds in *Adorybiotus coronifer* during exposure to desiccation was accomplished by Westh and Ramløv, who showed that time spent in cryptobiosis was ignored by the internal clock of the animals and inanimation produced a time shift in the age of tardigrades [47]. 

To explain anhydro-, cryo- and other forms of cryptobiosis, research groups examined colligative properties of solutes in water [36, 48, 49]. At present, it is known that trehalose and other polyhydroxy compounds by themselves are not sufficient to induce cryptobiosis, and heat shock and cold shock proteins expressed in organisms *viz*, the resurrection plant [50], *Artemia* cysts [51, 52, 53], bacteria [54, 55], and tardigrades [56, 57, 58] could also aid survival during desiccation and other stresses. Consequently, "water replacement" and other hypotheses (discussed below) were forwarded and argued to explain influence of solutes on the formation of ice mixtures, and stabilization of cellular structures which protect organisms in cryptobiosis.

## Structure, Properties, and Applications of Trehalose

### Structure and properties

**Figure 1:**
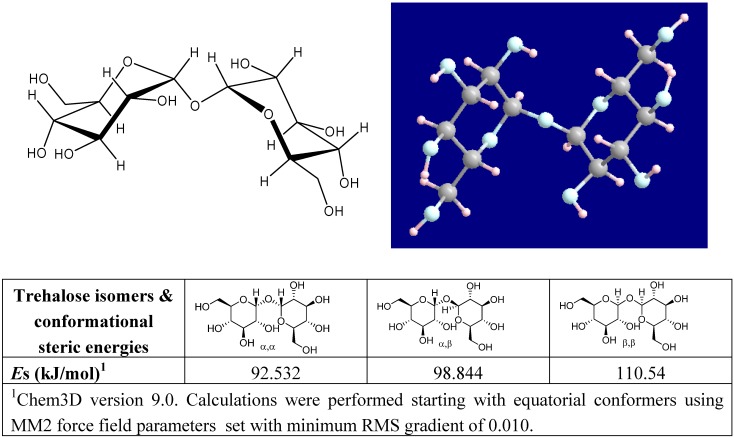
Structure of α,α-trehalose with molecular modeling of trehalose optimized by MOPAC PM3 calculation; and an assessment of the conformational steric energies of all possible trehalose isomers.

Naturally occurring trehalose is α-d-glucopyranosyl-(1→1)-α-d-glucopyranoside [59], whose dihydrate crystal structure was solved by Taga *et al.* [60]. It has the lowest energy conformation among the three isomers (α,α−, α,β−, and β,β−) that are possible for trehalose (Figure 1). The steric energy of each conformer was readily verified by us upon modeling all trehalose diastereomers with MM2 force field parameters set with minimum RMS gradient of 0.010 in Chem3D (CambridgeSoft, Inc.). The disaccharide is non-reducing because acetals of two glucose moieties are linked via a 1,1-glycosidic ether and are unavailable for reduction. Trehalose is thermodynamically and kinetically the most stable non-reducing natural disaccharide. The bond energy of the glycoside oxygen, bonding the two hexoses at C1 in trehalose, is very low (less than -4.2 kJ mol^-1^), and in sucrose it is +113 kJ mol^-1^ [61]. Consequently it is easy to explain why:

- 4% Aqueous trehalose solutions resist degradation between pH 3.5 and 10, at 100°C (24 h);
- The sugar is unreactive with amines, amino acids, and proteins (Maillard reaction); and
- Unlike sucrose, trehalose resists caramelization (browning) in prepared foods, and can be used as an excellent food bulking agent.

In addition to the above, its biological and reactivity features make trehalose very attractive for *de novo* explorations in clinical, pharmaceutical, immunological, and polymer research. 

Important properties to encourage industrial uses of trehalose are summarized in Table 1 [62]. Melting points and heats of fusion are different for the dihydrate and anhydride crystals because water lowers the melting point and increases heat consumption during fusion. Trehalose is very soluble in water and in aqueous ethanol, and is readily crystallized from solutions comprising >80% ethanol [3].

**Table 1:** Properties of α,α-trehalose (reproduced courtesy of IUPAC and Hayashibara Biochemical Laboratories [62]).

**Melting point**	dihydrate	97.0°C
	anhydride	210.5°C
**Heat of fusion**	dihydrate	57.8 kJ mol^-1^
	anhydride	53.4 kJ mol^-1^
**Solubility**	68.9 g/100 g H_2_O at 20°C	
**Optical rotation**	[α]_d_ +178°	
**Relative sweetness**	45% of sucrose	
**Digestibility**	digested and absorbed by the small intestine	
**pH stability of solution**	> 99% (pH 3.5-10, at 100°C for 24 h)	
**Heat stability of solution**	> 99% (at 120°C for 90 min)	

### Mechanisms of phospholipid and protein stabilization by trehalose

Trehalose is studied extensively in industry and academia due to the stabilizing and protective roles it plays in conjunction with other bio-molecules in organisms to enable them to withstand extreme environmental conditions. To explain cryptobiosis, Crowe and Clegg proposed and established the "water replacement hypothesis" [29, 33, 40, 45, 46, 63] and showed that increased concentrations of polyhydroxy compounds (glycerol, sucrose and trehalose) in cells permitted organisms to tolerate desiccation. Webb showed that addition of polyhydroxy compounds protected non-adapted bacteria against desiccation [64]. With molecular modeling studies, Warner showed that certain polyhydroxy compounds could fit within the hydration lattices of proteins and altered phase transition behaviors during cryptobiosis [65]. 

Through proton nuclear magnetic resonance (NMR) relaxation times studies, Clegg and colleagues revealed the hydration-dependent changes that occurred within *Artemia* cysts and supported their findings (and interpretations) with water sorption isotherms (by differential scanning calorimetry, DSC); quasi-elastic neutron scattering; and isothermal frequency scans [by dynamic mechanical and dielectric analyses, DMA and DEA, respectively, 40, 62, 66].

Clegg showed that values of spin-lattice and spin-spin relaxation times and self-diffusion coefficient (T_1_, T_2_, and D, respectively, units: msec, and 10^-6^ cm^-2^/sec) of protons of water in *Artemia* cysts changed with changes in molecular organization as systems stabilized themselves under different stresses. The T_1_, T_2_, and D values of protons in these systems showed narrow hysteresis behavior; and T_1_ and T_2_ reached minima when water concentrations of ca. 0.25 H_2_O g/g dry mass (for T_1_) and 0.15 g/g (for T_2_) were reached. These observations suggested a phase change, i.e., near cessation of rotational and translational motion at those concentrations and stopping of metabolic activities in cysts. Below the critical concentrations, water was suggested to be free to move near the primary hydration sphere (observed by increases in T_1_, T_2_ and D); and above the critical concentration T_1_, T_2_, and D values increased with increasing motion of water in bulk.

Clegg *et al.* explained these observations with the suggestion that polyhydroxy compounds can replace water at the surfaces of membranes, in the bulk phase, and within cells to permit formation of amorphous ice instead of ice crystals [40]. Furthermore, since amorphous ice has no sharp edges, the organism remain protected during phase changes. They further corroborated their suggestions by revealing similar changes in phase and NMR relaxation times for water trapped in bovine serum albumin (BSA) powders in the presence of glycerol [63]. 

Crowe *et al.* investigated the effects of polyhydroxy compounds, especially trehalose on phospholipids in membranes and liposomes [25, 67, 68, 69, 70, 71]. Regardless the nature of lipid, lipoprotein, or membrane they studied, Crowe and colleagues showed that stabilization of lipid systems with trehalose enabled the membranes to retain both their performance properties (calcium transport, ATPase activity, etc.) and contents within [67]. Furthermore they found that structural integrity and activities of cellular membranes and liposomes were best preserved at 20% trehalose by weight, which is similar to trehalose concentrations in anhydrobiotic nematodes. 

Among 11 polyhydroxy compounds tested by Crowe and colleagues, trehalose and sucrose appeared statistically comparable. However, trehalose exhibited highest effectiveness in assays involving ^3^H_2_O and fluorescent dye retention, and isocitrate dehydrogenase activity in systems stabilized by trehalose before and after freeze-drying [69, 70, 71]. By investigating the infrared P=O vibrational frequency (at ca. 1200-1250 cm^-1^), the authors explained that trehalose stabilized phospholipids at low water concentrations by replacing water and forming stable hydrogen bonds between the hydroxyl (trehalose) and phosphate groups (phospholipids) [25, 68, 72]. This model is supported by other experiments: surface pressure-area isotherms, surface potential-area isotherms, and molecular modeling [73, 74]. 

After a brief interlude in the literature, other reports attributed conformational fluctuations within macromolecules and lipids in membranes to changes in phase, and advised that glassy sugar matrices hindered ice crystallization and phase separation in cytoplasm [75, 76, 77, 78, 79, 80]. They further added that in the vitreous state, amorphous ice and solutes protected essential intracellular components from deleterious interactions with sharp edges of ice crystals, and macromolecules and organelles embedded in sugar glass matrices were trapped in space and time since molecular diffusion and chemical reactions had halted (the vitrification hypothesis). Evidence for this in anhydrobiotic cells came from studies on corn embryos by DSC [81]. 

**Figure 2:**
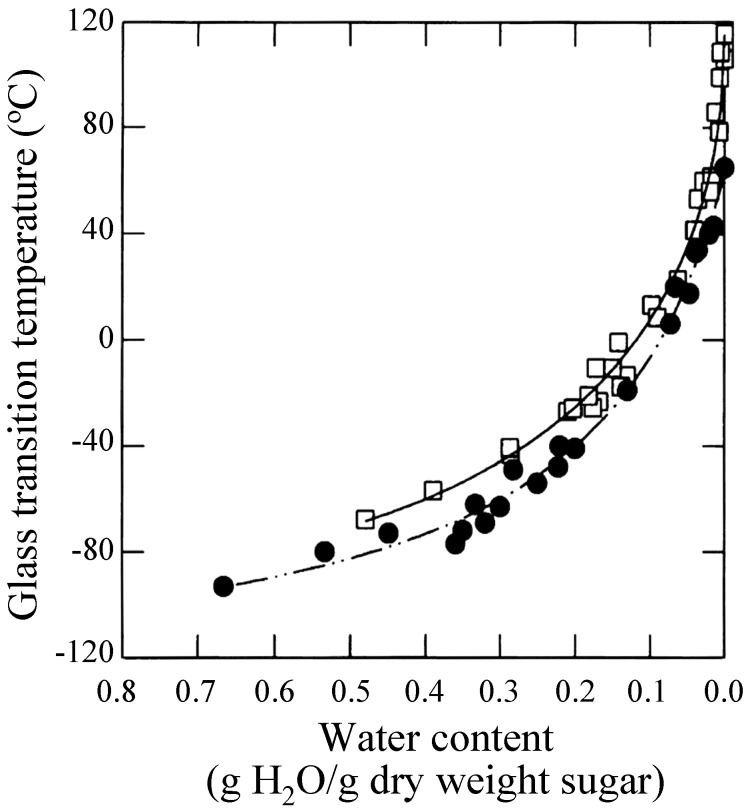
State diagrams for *T*_g_ of sucrose (closed circle) and trehalose (open square), reproduced courtesy of Professor L. M. Crowe and the Biophysical Society [84].

Sun *et al.* showed that loss of long-term viability in seeds at given water content occurred above the glass transition temperature (*T*_g_) of glass matrices [82]. Green and Angell reported that the trehalose-water system is unique and called it the "trehalose anomaly". They noted higher *T*_g_ at all water contents in trehalose-water systems (in the order of *T*_g_: trehalose > maltose > sucrose > glycerol) and that maximum distinction was observed when the stoichiometry of water to the glucose ring was 1:1 [83]. This is supporting the Crowe’s state diagrams for the trehalose-water system and the sucrose-water system wherein Crowe showed that the *T*_g_ for trehalose was higher than that for the sucrose system (Figure 2) [84]. 

Sun *et al.* also revealed that liposomes stored in sugar glasses showed extremely slow leakage of trapped solutes below *T*_g_, and the leakage rate increased rapidly when the temperature exceeded *T*_g_. Furthermore the major factor for solute leakage was attributed to vesicle fusion [85].

While many discussions continue on the two hypotheses (water replacement and vitrification), they are not (and cannot be) mutually exclusive [27, 33, 72, 84, 86]. Vitrification occurs upon interaction of membranes, intracellular components, macromolecules, water and sugars; some water is replaced by the polyhydroxy compounds (sugars) in the vitreous state; and the phenomena preclude formation of large ice crystals that are deleterious to the cellular membranes and machinery.

Interestingly however, recent molecular dynamic (MD) simulations revealed new insights. Sum *et al.* [87] and Pereira *et al.* [88] independently showed with MD simulations that direct interactions of trehalose and phosphates in phospholipid bilayers occurred through hydrogen bonds, and that these interactions were enhanced at higher temperatures. The results supported the water replacement hypothesis even though water molecules at bilayer surfaces were not completely replaced in simulations [88]. 

On the other hand, when interaction of trehalose with carboxymyoglobin (MbCO) and lysozyme were studied by MD simulations, trehalose was found not to interact with the proteins through hydrogen bonds [89, 90, 91]. Cottone *et al.* showed that motional freedom of MbCO embedded in trehalose-water matrix was restricted as if the MbCO molecule was kept at low temperatures, or as if it was trapped in a highly viscous system [89]. In addition, studies with lysozyme simulations by Lins *et al.* showed that while trehalose molecules clustered and moved toward lysozyme, coating by trehalose did not significantly reduce the conformational fluctuations in lysozyme [91]. Furthermore, they contrasted these observations with the interactions of trehalose and phospholipids, and noted that few trehalose molecules bound to MbCO [90]; and no trehalose molecules formed hydrogen bonds with lysozyme [91]. They proposed that effective protection of proteins by trehalose was due to proteins embedding in glasses of polyhydroxy compounds as if they were mechanically entrapped and unable to move within the glassy matrix, but able to change conformations at the same location.

More recently, yet another hypothesis was proposed based on DSC [92, 93], Raman and neutron diffraction [94, 95], and fluorescence [96] studies. This hypothesis explained a kosmotropic effect of sugars. That is, trehalose destroys the tetrahedral H-bond network of water, rearranges water molecules about biological structures; and accordingly reduces the amount of water at the interface of biomacromolecules and membranes, which would prevent structural fluctuations and damages by water during freezing and/or drying. 

While results of MD simulations of proteins with trehalose are explained by different hypotheses delineating nuances, none eliminates either or both the water replacement and/or vitrification hypothesis, and strengthens the notion that there are overlaps among proposed hypotheses. 

### Protection of proteins by trehalose

The protective effects of polyhydroxy compounds on proteins was explained 80 years ago by Beilinsson, who found that sucrose inhibited heat denaturation of ovalbumin [97]. After that, evidence accumulated sporadically between the 1960s and early 1980s [98, 99, 100, 101, 102, 103, 104, 105]. Later it was expanded to explain protein, organelle, and organism stabilization by other sugars and polyhydroxy compounds; and still later concepts were merged to explain anhydrobiosis [71, 106].

Freeze-drying of protein solutions is thought to be harmful to protein structure and function, and yet it is a very convenient method that allows long-term preservation of proteins without loss of activity. Hanafusa studied the effects of sucrose and glycine additives on freeze-drying and freeze-thawing of catalase. He found that addition of 30 mM aqueous sucrose to catalase prior to freeze-drying resulted in 85% retention of original enzyme activity, in contrast with 13% recovery of activity without additives [101]. Carpenter and Crowe studied the stabilization of phosphofructokinase with sugar during freeze-drying [71, 107]. While the activity of phosphofructokinase is lost when the protein is freeze-dried without additives, 60% to 80% of enzyme activity is retained when the protein is freeze dried in the presence of high concentrations of polyhydroxy compounds. Disaccharides were found to be better at protein preservation than monosaccharides; and trehalose and mannose were marginally more effective than sucrose. Since then, studies have revealed that trehalose protected proteins from denaturation during freezing, drying, freeze-drying, and heating [96, 107, 108, 109, 110, 111, 112, 113, 114, 115]. 

To summarize, the three proposed mechanisms for the protection of proteins by trehalose are (described above):

(a) The direct interaction between trehalose molecules and proteins through hydrogen bonds (water replacement hypothesis);
(b) The trapping of water molecules close to protein surface (water-layer hypothesis); and
(c) The entrapment of proteins conformations in high viscosity trehalose glasses (mechanical-entrapment hypothesis) [91].

In addition, new developments claim new roles wherein trehalose works as a chaperone in protein stabilization and protection [110, 113, 116, 117, 118]. It is also becoming clear that trehalose can reduce aggregate formation of amyloid proteins [119, 120, 121, 122, 123, 124, 125] and the oral administration of trehalose reduced polyglutamine aggregates and increased the survivability of transgenic mice with Huntington's disease [120]. 

Based on the known and emerging roles, it is easy to speculate that trehalose is a promising lead in understanding diseases related to protein complexation and aggregation, and in the discovery of useful therapeutic agents.

### Protection of mammalian cells by trehalose

Insights from investigations on the protective roles of trehalose in cells have spawned an industry on cryopreservation technologies. At present, cells including sperm cells and oocytes, tissues, and organs for use in human and veterinary medicine employ cryopreservation in diagnostic and clinical applications. Hereafter, long-term preservation of dried viable human cells at room temperature is a new and valuable research objective [126, 127, 128]. 

It appears that polyhydroxy compounds are needed on both sides (in and out) of cell membranes [129] for cells to be preserved for long periods. This notion emerged from observations on bakers' yeast *Saccharomyces cerevisiae* that became resistant to drying after trehalose accumulated both inside and outside the cells [130, 131].

With this insight, Beattie *et al.* (Crowe's group) introduced trehalose to pancreatic Langerhans cells using a leaky state in membranes generated by thermotropic transitions. Cryosurvival of these pancreatic cells was longest when the intracellular concentration of trehalose was 2.0 M in aqueous dimethylsulfoxide (DMSO) [132]. 

Toner's group found that the long-term post-thaw survival rates of cryopreserved mouse fibroblasts and human keratinocytes were improved > 80% and 70%, respectively, using 0.2 M aqueous trehalose inside and outside the cells, and they introduced trehalose intracellularly using a genetically engineered *Staphylococcus aureus* protein, which formed reversible 2-nm transmembrane pores in the lipid bilayers [133]. 

Simultaneously, Levine's group reported that human primary fibroblasts expressing trehalose could be maintained in the dry state for up to five days [134]. They introduced *Escherichia coli otsA* and *otsB* genes that encode enzymes for trehalose biosynthesis, using a recombinant adenovirus vector. Thereafter, Toner's group reported the preservation of mammalian cells under desiccation conditions using *Staphylococcus aureus* protein [135, 136]. Furthermore Crowe's group revealed that human platelets loaded with trehalose could survive freeze-drying [137] and trehalose was introduced to the platelets by simple incubation.

Other studies since have also confirmed the effectiveness of trehalose in cryopreservation or drying and preservation of mammalian cells, and these examples include: primary human hepatocyte cryopreservation in the presence of trehalose [138, 139], microinjection technique to introduce trehalose in failed-to-fertilize human oocytes followed by cryopreservation [140] and preservation of red blood cells [141], hematopoietic cells [142, 143], embryonic stem cells [144, 145], mitochondria [146], adipose cells [147]. 

The insights were later extended to corneal epithelia under desiccation [148]; it appears that trehalose solutions could be used to treat the dry eye syndrome [149] and advances continue.

Levine's group has shown yet another way to achieve enhanced desiccation tolerance in human cells without additives [150], and has suggested that there exist several procedures that can permit preservation human cells in the dry state. So it appears that possibly a combination of these procedures may be employed by industry to enhance cell preservation and survival for uses in human and veterinary medicine.

### Organ preservation and applications in diagnostic medicine

Trehalose is used in organ preservation, especially when extended periods > 24 h are needed. Wada's group developed the "ET-Kyoto solution" containing trehalose ("E" = extracellular and "T" = trehalose) for organ preservation [151]. They adjusted buffers and sodium and potassium levels in intra- and extracellular "new ET-Kyoto solutions" and enabled canine lung preservation for > 30 h without affecting the performance of endothelial cells and vasculature upon perfusion [152]. Newer ET-Kyoto solutions contain *N*-acetyl-cysteine, dibutyryl adenosine 3',5'-cyclic monophosphate and nitroglycerin, and find use in the preservation of the trachea, kidney, skin/muscle flap, amputated digits, pancreas, and the liver [153, 154]. 

Trehalose has also gained importance in the prolonged stabilization and preservation of vaccines, antibodies, and diagnostic kits [155, 156, 157, 158, 159, 160, 161], because these medicinal agents must sometimes be carried long distances [162]. The mechanism for stabilizing vaccines and antibodies is the same as that for membranes and proteins. Dry blood-group-typing plates that contain monoclonal antibodies also include small amounts of trehalose to enable indefinite storage of reagents at room temperature [161].

### Uses of trehalose in functional foods, flowers, cosmetics, and other applications

Efficient industrial production of trehalose (see below) has greatly expanded the range of possible applications for the disaccharide [163]. Trehalose is now consumed in the food industry as a sweetener and bulking agent when less sweetness than that provided by sucrose is needed. It masks unpleasant off-tastes and malodors, and preserves starch, lipids, and proteins against degradation by oxidation, heat, and cold [61, 62, 108, 164]. 

Kubota *et al.* found that trehalose was complexed with Ca^2+^ and Mg^2+^ ions to yield crystals and confirmed complexation by NMR [164]. Furthermore, they found that trehalose prevented the Ca^2+^ precipitation by phosphate ions and Mg^2+^ losses from treated vegetables and meat. 

Nishizaki *et al.* found that trehalose administered orally in ovariectomized mouse models suppressed progress of osteoporosis, and suggested that trehalose suppressed excessive osteoclastogenesis by inhibiting differentiation of osteoclasts, which was induced by secretion of interleukin-6 in bone marrow [165, 166, 167]. Otsubo and Iwaya-Inoue further extend the applications with the finding that 0.1 M trehalose in water delays senescence in cut flowers [168]. Furthermore trehalose is used in the cosmetic industry, because it acts as a moisturizer and stabilizers for liposome contained in cosmetics and for lipids and proteins in skins [62, 163]. 

## Production of Trehalose

### Chemical synthesis

β,β-Trehalose (termed *iso*-trehalose) was the first isomer synthesized chemically in 1909 [169]. Fischer and Delbrück synthesized it in 13% yield upon treatment of 2,3,4,6-tetra-*O*-acetyl-β-d-glucose with phosphorus pentoxide [170]. They also found that it was obtained as a byproduct in 5% yield in the preparation of 2,3,4,6-tetra-*O*-acetyl-α-d-glucopyranosyl bromide. An improved preparation of octa-*O*-acetyl-β,β-trehalose was achieved by Helferich and Weis in 1956, who effected preparation in 31.5% yield with 2,3,4,6-tetra-*O*-acetyl-β-d-glucose and 2,3,4,6-tetra-*O*-acetyl-α-d-glucosyl bromide in nitromethane using mercuric cyanide [171]. 

Chemical synthesis of α,β-trehalose (termed *neo*-trehalose) was first claimed in 1928 by Vogel and Debowska-Kurnicka who obtained a substance, m.p. 68-70°C, [α]_D_ +68.1 (chloroform), by condensing 2,3,4,6-tetra-*O*-acetyl-d-glucose in toluene in the presence of zinc chloride and phosphorus pentoxide (yield: 15%) [172]. Though the reported specific rotation of the product corresponds to that reported by some later workers, the melting point as the octaacetate form was low. Sharp and Stacey obtained octa-*O*-acetyl-α,β-trehalose by shaking 2,3,4,6-tetra-*O*-acetyl-α-d-glucosyl fluoride with silver carbonate, Anhydron, and iodine in chloroform in good yield (40%) [173]. 

**Figure 3:**
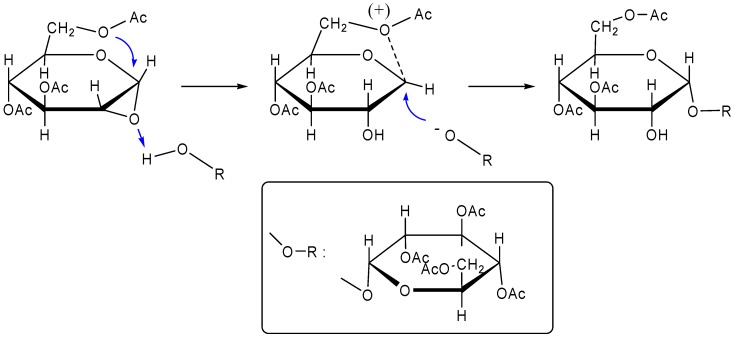
Chemical synthesis of α,α-trehalose by the method of Lemieux and Bauer [175].

α,α-Trehalose (also "trehalose"), the natural isomer and only isomer biosynthesized in organisms, remained elusive in early 1940s. Frahm reported a 31% yield of octa-*O*-methyl-α,α-trehalose by heating 2,3,4,6-tetra-*O*-methyl-d-glucose with concentrated hydrochloric acid [174]. In 1954, the definitive chemical synthesis of α,α-trehalose was reported by Lemieux and Bauer [175]. They synthesized the isomer by heating of 2,3,4,6-tetra-*O*-acetyl-d-glucose (containing about 65% of the α-d-anomer) with 3,4,6-tri-*O*-acetyl-1,2-anhydro-d-glucose (Brigl's anhydride) in benzene (Figure 3). Just prior to them, Montgomery and Weakly had obtained octa-*O*-acetyl-α,α-trehalose by acetylation of cornstarch hydrolyzed with acid catalyst [176]. The chemical synthesis of α-d-glucosidic linkages appears not to be so simple, compared to enzymatic reactions, nonetheless, α,α-trehalose was obtained as a byproduct in the synthesis of 3-*O*-α-d-glucopyranosyl-d-glucose [177]. 

Recently, Haines synthesized l-trehalose by reaction of 2,3,4,6-tetra-*O*-benzyl-l-glucose with trimethylsilyl trifluoromethanesulphonate in dichloromethane in the presence of a special molecular sieve SYLOSIV^®^ A4 and generated the α,α-isomer preferentially [178]. He then investigated the stabilizing effect of l-trehalose on alkaline phosphatase in comparison with d-trehalose. Both isomers provided some protection effect on freeze-drying and heating. However, the effectiveness of d-trehalose was greater than that of l-trehalose, and the author attributed the result to the water replacement hypothesis and suggested that there might be a chiral bias in proteins that enabled them to interact differently with d- and l-trehalose [179]. To enhance understanding of disaccharide protein recognition phenomena, Bertozzi and colleagues prepared mono- and dideoxygenated α,α-trehalose analogs and completely characterized them by NMR and intend to use them to probe substrate protein interactions of trehalose derivatives with mycobacterial sulfotransferase [180]. 

### Biosynthesis

**Figure 4:**
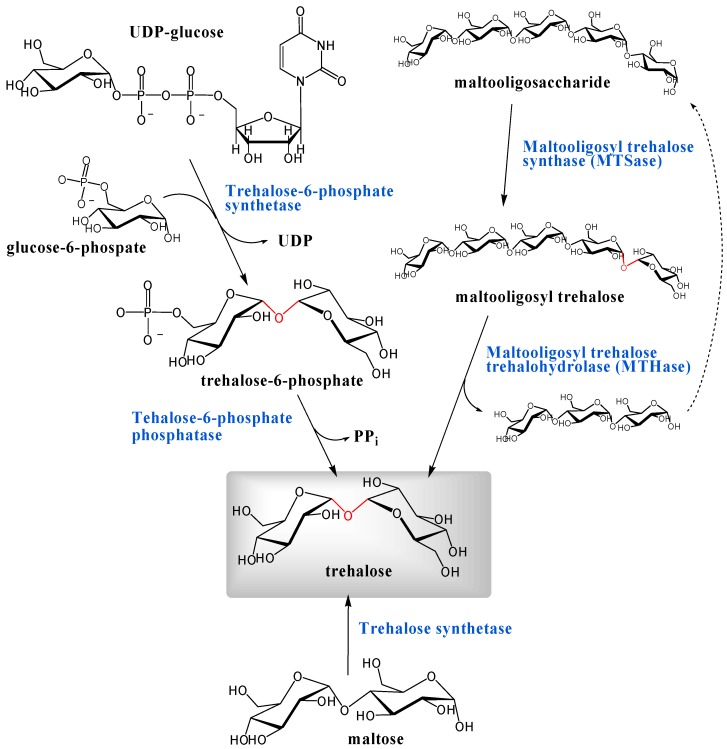
Three pathways for biosynthesis of trehalose. α,α-1,1-Glycosidic linkages are indicated in red.

There appear to be at least three different pathways that lead to the biosynthesis of α,α-d-trehalose and the most well-known among them is via trehalose-6-phosphate (Figure 4). Trehalose phosphate synthase catalyzes the reaction of UDP-glucose or GDP-glucose with glucose-6-phospate to produce trehalose-6-phosphate, which is hydrolyzed by the phosphatase to trehalose (see below). Leloir and Cabib demonstrated this biosynthesis in brewers' yeast [181, 182]. They purified UDP-d-glucose:d-glucose 6-phophate 1-d-glucosyl transferase (EC 2.4.1.15) which catalyzed the reaction of UDP-glucose with glucose-6-phosphate to provide trehalose-6-phosphate. The enzyme showed maximum activity at pH 6.6 in the presence of 25 mM of Mg^2+^. The reaction involving UDP-glucose was also found in numerous other organisms, including locusts, silkmoths, *Mycobacterium tuberculosis*, *Moniliformis dubius* and *Dictyostelium discoideum* [3]. Similar enzymes coupling other d-glucosyl donors also exist. Elbein demonstrated that *Streptomyces hygroscopicus* used only GDP-d-glucose as a d-glucosyl donor rather than UDP-d-glucose and isolated trehalose-6-phosphate synthase (GDP-d-glucose:d-glucose 6-phosphate 1-d-glucosyl transferase, EC 2.4.1.36) from cell-free extracts of *Streptomyces hygroscopicus* [183]. In addition to *Streptomyces hygroscopicus*, other *Streptomyces* species also converted GDP-d-glucose [3]. In addition, trehalose-6-phosphate synthase from *Mycobacterium smegmatis* was able to utilize ADP-d-glucose, CDP-d-glucose, TDP-d-glucose, GDP-d-glucose, and UDP-d-glucose to produce trehalose-6-phosphate at different rates relative to substrates [184]. 

**Figure 5:**
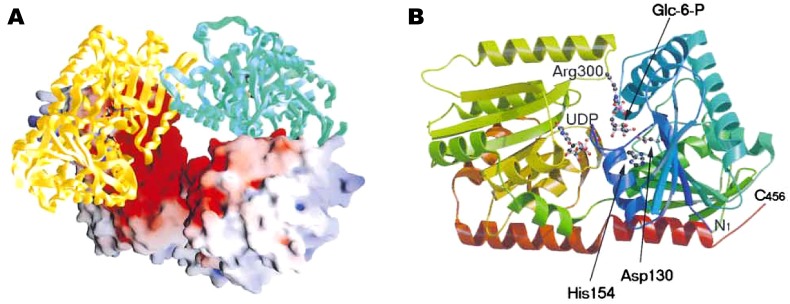
Crystal structure of trehalose-6-phosphate synthase (OtsA) provided by Gibson *et al*. [185]. (A) Tetramer of the enzyme. The electrostatic surface representation (positive, blue; negative, red) and the protein cartoon figure are mixed. (B) Ribbon representation of the structure of OtsA. The two ligands, glucose-6-phosphate and UDP, are shown in ball-and-stick model. Figure courtesy of Professor G. J. Davies and Cell Press.

The three-dimensional structure of trehalose-6-phosphate synthase from *Escherichia coli* was provided by Gibson *et al*. [185, available at the Macromolecular structures database with accession code 1GZ5, PDB] (Figure 5).

**Figure 6:**
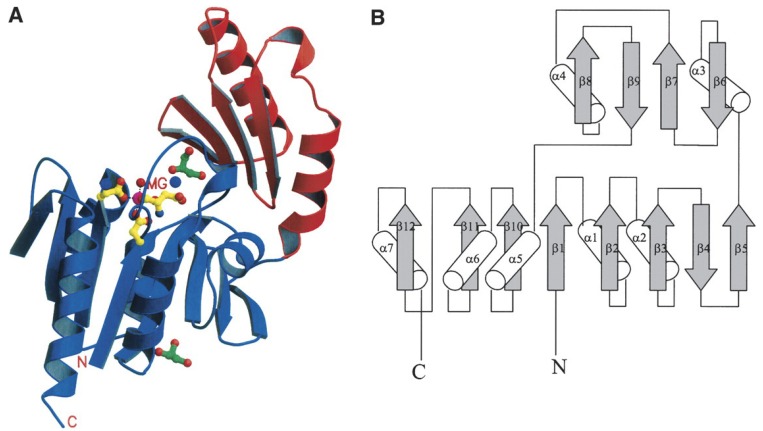
Crystal structure of trehalose-6-phosphate phosphatase provided by Rao *et al*. [188]. (A) Ribbon representation of structure of the enzyme (hydrolase domain, blue; cap domain, red). The magnesium ion (magenta), its coordinating residues (yellow), the two sodium ions (purple), and the two bound glycerol molecules (green) are shown in ball-and-stick model. (B) Topology diagram for the secondary structure of the enzyme (strands, arrows; helices, cylinders). Figure courtesy of Professor S. Swaminathan and Protein Science.

The next step in the biosynthesis of trehalose is conversion of trehalose-6-phosphate to trehalose by trehalose-6-phosphate phosphatase (EC 3.1.3.12), which involves release of inorganic phosphate and requires a divalent cation, preferably Mg^2+^, for activity [1]. Leloir and Cabib detected its activity in their experiments on the biosynthesis of trehalose-6-phosphate in brewers' yeast [182]. Friedman [186] and Matula [187] purified trehalose-6-phosphate phosphatase from extracts of *Phormia regina* and *Mycobacterium smegmatis*, respectively. The enzyme from *Phormia regina* was highly specific for trehalose-6-phosphate among a variety of sugar phosphates tested, however, it showed 8% activity for the hydrolysis of d-glucose-6-phosphate compared to trehalose-6-phosphate. The enzyme from *Mycobacterium smegmatis* was highly specific and also had slight activity on d-mannose-6-phosphate and d-fructose-6-phosphate. The three-dimensional structure of trehalose-6-phosphate phosphatase from *Thermoplasma acidophilum* was provided by Rao *et al*. [188, available at the Macromolecular structures database with accession code 1U02, PDB] (Figure 6).

The genes encoding the two enzymes are located in one operon in *Escherichia coli*: *otsA* gene encodes trehalose-6-phosphate synthase; and *otsB* gene encodes trehalose-6-phosphate phosphatase. They were cloned in 1992 [189]. Cloning and mutant analysis showed that the *otsBA* genes constitute an operon with *otsB* proximal to the promoter [189, 190]. These genes are transcriptionally activated both by osmotic stress and by growth into the stationary phase. The *otsBA* operon is located at the co-ordinate 1992 kb (42 min in the map position) on the physical map of the *Escherichia coli* chromosome. The corresponding genes of yeast, *Saccharomyces cerevisiae*, were cloned in 1992 and 1993 [191, 192]: *tps1* gene encodes trehalose-6-phosphate synthase; and *tps2* gene encodes trehalose-6-phosphate phosphatase. These proteins are present as a part of the protein complex built from four subunits [193]. Other two proteins, TSL1 (coded by *tsl1* gene) and its homologue TPS3 (coded by *tps3* gene), are thought to be regulatory proteins. However, besides this complex, a significant part of trehalose-6-phosphate synthase is thought to be present in the cell as free monomeric protein [193]. 

*Escherichia coli* genes were incorporated into human cells to enhance its viability under dry conditions described above [134]. Transfection of *Escherichia coli otsA* and *otsB* genes or the yeast *tps1* gene into higher plants, tobacco plants, also showed improvements in drought tolerance [194, 195].

Most recently, expression experiments of these genes in higher plants revealed that they regulate cell shapes and functions [195, 196, 197, 198, 199, 200]. For example, *otsA* and *otsB* affects growth and development (pleiotropic effects) in Arabidopsis and the effects depend on increased trehalose-6-phosphate, rather than increased trehalose accumulation, which indicates that trehalose-6-phosphate may be involved in cellular signaling [197].

Alternate pathways for trehalose biosynthesis emerged from bacterial research by researchers in Hayashibara Biochemical Laboratories, Inc. One pathway involves trehalose synthase enzyme, firstly found in *Pimelobacter* sp., which catalyzes rearrangement of maltose wherein the α-1,4-linkage was converted to the α,α-1,1-glycoside [201]. Bacteria *Pimelobacter* sp. R48, *Pseudomonas putida* H262, isolated among approximately 2500 strains obtained from soil by Nishimoto *et al.*, showed the ability to produce trehalose from maltose. The responsible enzyme is referred as trehalose synthase (EC 5.4.99.16, maltose α-d-glucosyltransferase). Furthermore, Nishimoto *et al.* also purified a thermostable trehalose synthase from *Thermus aquaticus*, which was stable from pH 5.5 to 9.5 and up to 80°C for 60 min [202].

Other biosyntheses of trehalose, also discovered by the same group [203], generated trehalose from maltooligosaccharide and the trehalose producing soil bacterium was *Arthrobacter* sp. Q36. When the culture supernatant of this strain was incubated with 5% of maltopentaose in sodium phosphate buffer at 40°C, the trehalose content reached about 40% of total sugars within 8 h. The remnant sugar was maltotriose (60%). In the course of the reaction, an unknown sugar appeared and disappeared with an increase of trehalose. Maruta *et al.* characterized the transient unknown sugar by field desorption mass spectrometry (FD-MS), methylation analysis and ^13^C NMR [203] and revealed that the unknown sugar was α-maltotriosyl trehalose, indicating that the pathway consisted of two enzymatic reactions. In the first reaction, maltopentaose was transformed into α-maltotriosyl trehalose through a conversion of the α-1,4-glucosidic linkage to the α,α-1,1-glycoside; and the second reaction converted α-maltotriosyl trehalose to trehalose by hydrolysis of the α-1,4-glucosidic linkage between maltotriose and trehalose (Figure 4). The enzymes involved are: maltooligosyl trehalose synthase (MTSase, EC 5.4.99.15) [204] and maltooligosyl trehalose trehalohydrolase (MTHase, EC 3.2.1.141) [205]. 

Maruta *et al.* cloned the genes encoding the two enzymes from *Arthrobacter* sp. Q36 and found three genes, *treX*, *treY*, and *treZ*, were clustered in one operon (*treXYZ*) on the bacterial genome [206, 207], and the genes, *treX*, *treY*, and *treZ*, encoded isoamylase, MTSase, and MTHase, respectively. The operon *treXYZ* is important for the bacterial conversion of glycogen to trehalose. Isoamylase (EC 3.2.1.68, glycogen 6-glucanohydrolase, glycogen debranching enzyme) catalyzes the hydrolysis of the α-1,6-glucosidic linkage in glycogen. 

Nakada *et al.* of Hayashibara found that two hyperthermophilic acidophilic archaea, *Sulfolobus acidocaldarius* and *Sulfolobus solfataricus* also produce these enzymes [208] and showed that MTSase of *Sulfolobus acidocaldarius* was stable from pH 4.5 to 9.5 and up to 85°C for 60 min. Only a month after them, Kato *et al.* of Kirin Brewery reported a similar pathway in the cell homogenate of the hyperthermophilic acidophilic archaea, *Sulfolobus solfataricus* KM1 and purified the enzymes [209]. 

### Industrial production

Early preparations of natural α,α-trehalose from vegetable and fungal sources involved extraction with ethanol, liberation from protein by addition of lead (II) acetate, removal of lead by precipitation with hydrogen sulfide, filtration, and decolorization with carbon [169]. This was very costly and limited its utility, and consequently trehalose was only available for research and cosmetic use in small amounts, and was unavailable for food industry applications. In the early 1990s, the cost of 1 kg of commercial trehalose was estimated in the US to be > $200 [9, 210] and researchers were compelled to seek ways for industrial production [163]. 

Panek *et al.* developed fermentation methods to overproduce trehalose with yeast [9]. Haynie and Whiteside reported the cell-free enzymatic synthesis of trehalose with *in situ* generation of UDP-glucose and the cycle could be executed approximately 10 times [211]. Calgene, BTG International and MOGEN International investigated transgenic plants that produced trehalose [9, 210, 212, 213, 214]. Best results came from Hayashibara’s methodologies. They firstly produced MTSase and MTHase from *Arthrobacter* sp. Q36 (described above), and after purification of enzymes with SF (sterilizing filtration) and UF (ultrafiltration) membranes, trehalose was obtained conveniently in high yields by enzymatic saccharification of starch (Figure 6) [61, 164]. However, the enzymes from *Arthrobacter* sp. Q36 become inactive over 50°C (the best temperature for saccharification is 55°C). 

Afterwards, they found more stable enzymes from *Arthrobacter ramosus* S34 [215], they used them in industrial production solved the low productivity of the enzymes problem by mutating original *Arthrobacter ramosus* S34 *N*-methyl-*N*'-nitro-*N*-nitrosoguanidine (NTG) and culturing thriving colonies [164]. Further processing advantages came from using inexpensive starch from corn and tapioca; significant for environmentally benign materials based on carbon neutrality..

Hayashibara also improved production yields from starch, because MTSase and MTHase can not utilize all parts of liquefied starch. They thus attained over 85% yield from starch by combined use of the enzymes isoamylase, cyclomaltodextrin glucanotransferase (CGTase), glucoamylase and α-amylase for saccharification [164]. Consequently, they were able to reduce the production costs to about one hundredth of the original (from > $200 to < $3 per kg) and enhanced production to 31,000 tons in 2007 to support increasing demand. A new plant which can produce 43,000 tons/year is at presently being set up in Okayama, Japan. 

**Figure 7:**
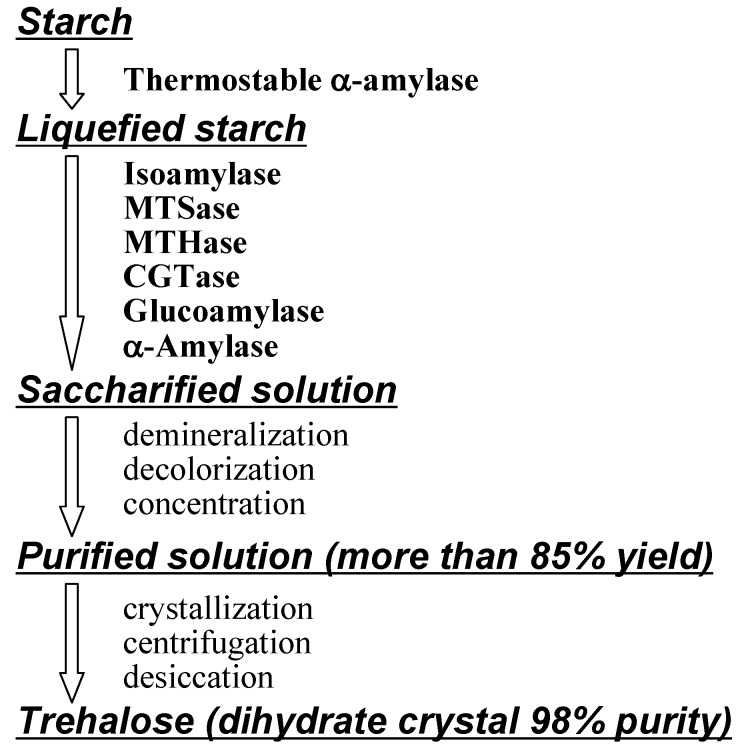
Industrial enzymatic production of α,α-trehalose (reproduced courtesy of IUPAC and Hayashibara Biochemical Laboratories [61]).

## Trehalose-based Polymers

### Network polymers

We propose herein that trehalose-based polymers should be classified in a different category of sugar-based materials involving the unique symmetrical and non-reducing disaccharide. This is because trehalose shows valuable physical chemical and biological properties due to its symmetrical structure, high thermodynamic and kinetic stability, that collectively influence material design, chemical synthesis, polymerization, polymer processing, material properties, and continued new application development. 

Despite the known advantages, trehalose-based polymers are hardly reported in the literature and it may have been due to:

(a) The price being very high (> $200/kg) until 1995 (now < $3/kg);
(b) It has no anomeric hydroxyl group whose reactivity is different from other hydroxyl groups; and
(c) The disaccharide is devoid of a galactosyl residue that shows affinity for interaction with cell receptors.

To change this paradigm, herein we describe the preparation of trehalose-based polymer networks, and then discuss the methods for trehalose-based linear polymers we have investigated. Accompanying our discussions on new materials development with trehalose, we will show the improved performance properties of our products.

Synthesis of trehalose-based polymer networks was straightforward, because trehalose has eight reactive hydroxyl groups. Using precedence from sucrose-based network polymers [216, 217, 218, 219] we adapted methods for use with trehalose and aimed for less trehalose substitution to lower preparation and processing costs. Patents on saccharide-based network polymer claiming applications with trehalose as a possible reagent also exist, and methods therein generate hydrogels for biomedical application [220, 221, 222]. 

While it may appear that the advantage of using trehalose in network polymers may be limited, since without enzymes its hydroxyl groups react non-specifically in synthesis, the disadvantage is mitigated by the exceptional chemical stability of trehalose in comparison with other sugars and polyols, and its thermosets bear potential to be hardy and low in cost in the construction of electronic instruments. 

**Figure 8:**
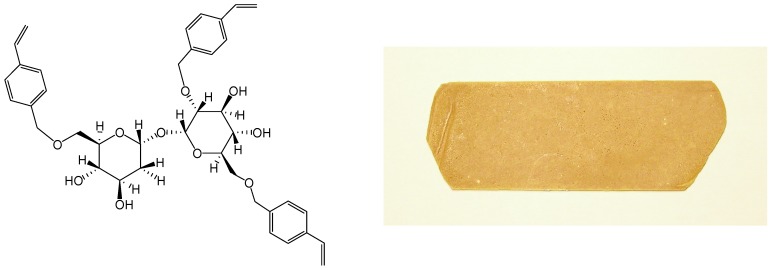
Typical structure of trehalose vinylbenzyl ether (left), and a photograph of the molded resin (right).

We started the synthesis of trehalose thermosets in 2001 and reported thermal curing of trehalose vinylbenzyl ethers (Figure 8) in 2004 [223]. Trehalose vinylbenzyl ethers were synthesized by reaction of trehalose with *p*-chloromethylstyrene in the presence of powdered sodium hydroxide and the average degree of substitution (DS) was kept between two and four by adjusting the *p*-chloromethyl-styrene feed ratio. The resin was a pale yellow powder at room temperature and liquefied beyond 80°C. The resins, DS 2.4 and 3.2, showed one exotherm at 132˚C and 191˚C respectively in DSC measurements, whereas the resins DS 2.8 and 3.0 showed two exothermal peaks indicating two-stage curing. Resins could be cured without initiator and molded into sheets by pressing at 30 kgf/cm^2^ (2.9 MPa) at 200˚C for 30 min in a hot press, followed by post-cure at 200˚C (30 min, Figure 8). Thermal decomposition temperature (*T*_d_) increased from 284˚C (trehalose) to ca. 330˚C upon substitution of hydroxyl groups; and glass transition temperature (*T*_g_, by thermomechanical analysis TMA) decreased from 143˚C to 118˚C with increase in DS. Thermal expansion coefficient, measured from 50˚C to 100˚C, was ca. 4 × 10^-5^ K^-1^. 

Thereafter, we prepared trehalose allyl ethers which were yellow liquids. The resin cured with cumene hydroperoxide at 100˚C, 1 h; 150˚C, 1 h; 170˚C 1 h (in a hot press); 200˚C, 12 h; and 220˚C, 2 h (electric oven) to give dark brown sheets (unpublished to date). Curing at high temperature in multiple steps was necessary because of the low reactivity and low viscosity of trehalose allyl ethers. Thermal decomposition temperature (*T*_d_) was around 320˚C for trehalose allyl ethers. Upon comparison, sucrose octa-*O*-allyl ethers performed just as well but used more allyl reactants per mole disaccharide and were clear colorless liquids [216, 217, 218, 219]. The discrepancy suggested that color in trehalose-based materials was due to incomplete reactions of allyl groups in the mixture which continue to oxidize in air.

**Figure 9:**
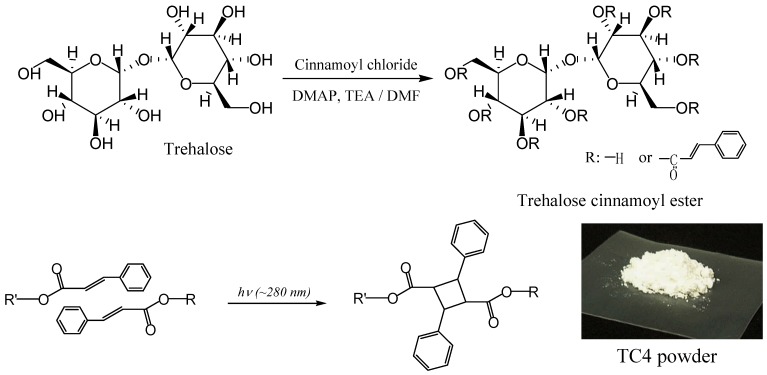
Synthesis of trehalose cinnamoyl esters and [2+2] Cycloaddition reaction of cinnamate groups (reproduced from the reference [224] with the permission of John Wiley & Sons).

Our next target was to generate polymer networks by photocuring so full advantage of trehalose’s inactivity towards Maillard reaction could be taken to generate efficient coatings for electronics equipment and optical materials with trehalose cinnamoyl esters (TC) [224]. Cinnamoyl moieties of different oxidation states (alcohols, aldehydes, acetals, acids, anhydrides, and esters) and different aryl substituents (hydroxyl, methoxy, aryloxy, and the like) are abundant at junctions of lignin and hemicellulose in plant cell walls. They offer exceptional opportunities for exploiting this natural resource for the development of environmentally friendly coatings. Furthermore, cinnamates contribute useful chemical and physical properties that ease polymer processing, such as: low material cost; photoreactivity of bis-conjugated carbon-carbon double bonds (by 2π+2π addition in both solution and solid states); in use durability of products; and environmental degradation when the materials are discarded after use (Figure 9) [225, 226, 227]. In addition, cinnamates are reported to afford photo-oriented materials [228, 229]. 

**Figure 10:**
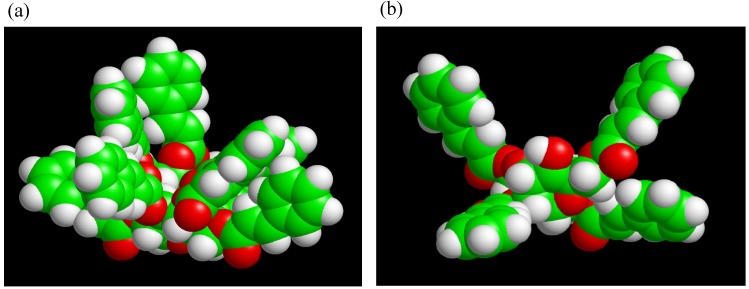
Molecular modeling of (a) TC8 and (b) TC4 optimized by MOPAC PM3 calculation (reproduced from the reference [224] with the permission of John Wiley & Sons).

Two different types of TC were synthesized using trehalose, cinnamoyl chloride, *N,N*-dimethyl-formamide (DMF), triethylamine and 4-(*N,N*-dimethylamino)pyridine. By changing the cinnamoyl chloride to trehalose feed ratios TC4 (lower DS), and TC8 (higher DS) monomers were obtained. UV and FT-IR spectra of TC changed within 5 min of UV irradiation, indicating that C=C bonds had reacted. Solubilities of photocured TC4 and TC8 films in chloroform had significantly decreased. The materials should be insoluble in any solvent if they are crosslinked. Rate of the spectral change for TC4 was faster than that for TC8, despite fewer cinnamoyl groups of TC4. Upon molecular modeling (Figure 10), we proposed that excess steric crowding and strained conformational arrangements of cinnamoyl substituents in TC8 could have prevented facile alignment of two excited cinnamoyl units during photoaddition to form cyclobutane and could have retarded the rates of TC8 derived networks. Furthermore, despite unreacted hydroxyl groups in TC4, the monomers were colorless, which validated our hypothesis why partially substituted trehalose allyl and vinyl benzyl ethers are colored and trehalose cinnamates are colorless. 

Transparent thin films of TC4 and TC8 could be prepared by casting their dichloromethane solutions on Petri dishes followed by UV irradiation with use of aluminum foil photomasks to create cured and uncured regions in the films. After irradiation, shapes of the photomasks were revealed in the masked (non-irradiated) regions by rapidly soaking films samples in the solvent to dissolve and remove uncured resin. 

Scanning electron microscopic (SEM) observations revealed that surfaces of photocured coating films were smooth and their edges had the characteristic features of swelling due to uneven evaporation of the solvent. We are testing these films for photoinduced orientation in our materials with linear polarized UV light.

### Linear Polymers

#### Polyaddition with diisocyanates

In comparison with trehalose-based polymer networks, and without using enzymes, the synthesis of trehalose-based bifunctional monomers for linear polymerization is challenging. Consequently they involve steps of protection and deprotection of hydroxyl groups to attain targets for linear structures as was observed with sucrose [230, 231, 232, 233]. 

Trehalose has two primary hydroxyl groups and six secondary ones and selectivity between these two types of hydroxyl groups is not controllable in non-enzymatic low cost reactions, so synthesizing bifunctional monomers from disaccharides for linear polymerization becomes cumbersome. However, if the selectivity in chemical reactions can be effectively tailored with trehalose, it becomes a very powerful tool for the design of bifunctional monomers because trehalose has a symmetric structure and it is thermally very stable. 

**Figure 11:**
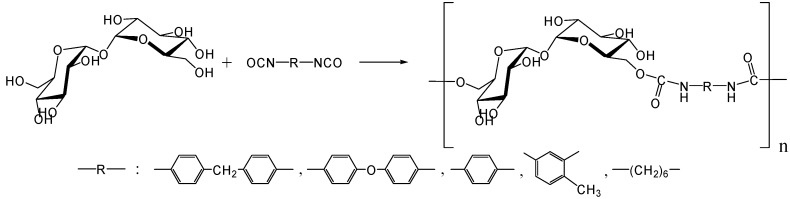
Synthesis of trehalose-based polyurethane.

**Figure 12:**
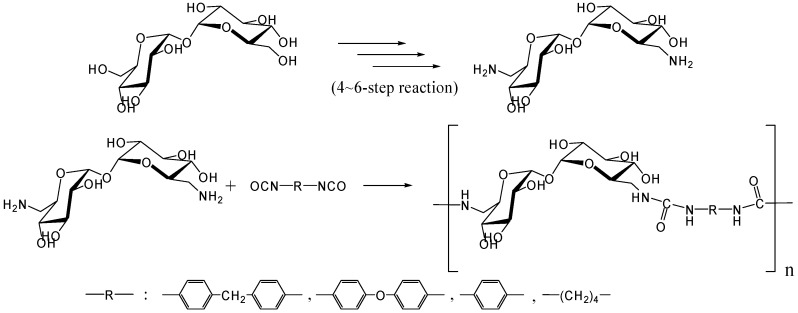
Synthesis of trehalose-based polyurea.

In 1979, Kurita *et al*. reported the synthesis of direct polymerization of trehalose with diisocyanates, which is the first trial to produce trehalose-based linear polymer (Figure 11) [234]. Though the idea is simple to execute, the selectivity in linear polymer formation was not clear as the authors could not rule out the possibility of chain branching. Fifteen years after the report, they achieved the polymerization of the diamino-type trehalose (6,6'-diamino-6,6'-dideoxy-α,α-d-trehalose) with diisocyanate to give a biodegradable polyurea containing trehalose units in the main chain (Figure 12) [235]. While the synthesis of the diamino-type trehalose required several steps, the polyurea was degraded not only by trehalase but also by α-amylase.

#### Enzymatic and chemoenzymatic synthesis

**Figure 13:**
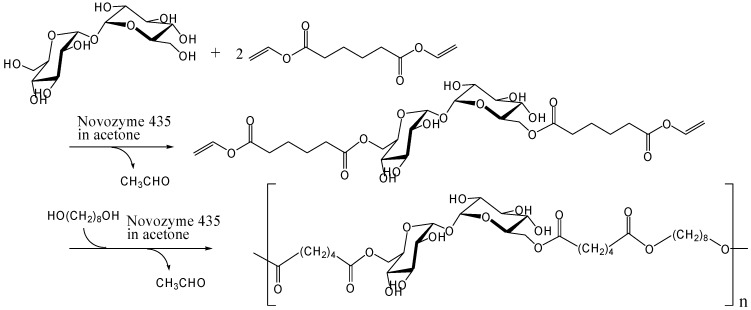
Enzymatic synthesis of trehalose-based polyester.

Dordick and co-workers investigated the enzymatic polymerization of trehalose to synthesize polyesters. Initially, they reported polyesters and polyesteramide containing sucrose in the main chain, and polyacrylate containing sucrose as a side chain [236, 237]. Prior to their work, Klibanov and coworkers showed that lipase and protease could catalyze the acylation of saccharides via transesterification [238, 239]. This enzymatic reaction is regioselective and it is reasonable to apply the reaction for synthesis of saccharide-based linear polymer. In 2000, Dordick and co-workers carried out the polymerization of trehalose 6,6'-*O*-divinyladipate with 1,8-octanediol in acetone using lipase B from *Candida antarctica* immobilized on an acrylic resin (Novozyme 435) (Figure 13) [240]. Trehalose 6,6'-*O*-divinyladipate was synthesized from trehalose and excess of divinyl adipate by Novozyme 435 (Figure 13). The enzymatic reactions occurred on the 6- and 6'-hydroxyl groups of trehalose. 

They adopted the two-step reaction, because one-step enzymatic polymerization of the disaccharide did not give polyesters with high molecular weight [236]. The weight average molecular weight (*M*_w_) of the products in the two-step reaction was 17000 with a polydispersity of 1.2. While the molecular weights are sufficient for self-standing polymers, they are not large for affording polymers with high moduli.

**Figure 14:**
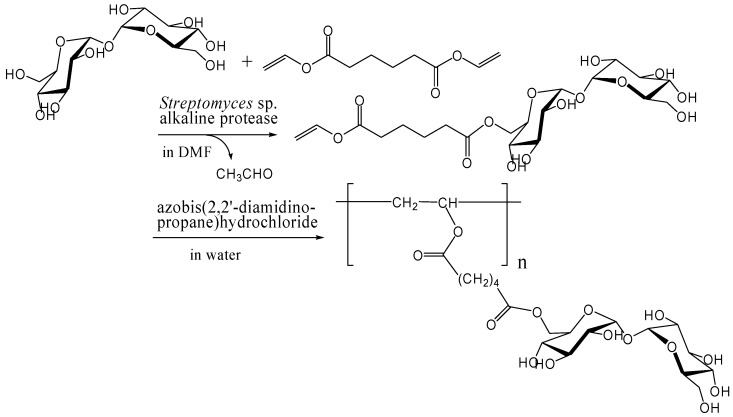
Chemoenzymatic synthesis of vinyl polymer containing trehalose in side chains.

Kitagawa and Tokiwa's group has found that monoacylation of trehalose with divinyl adipate selectively occurred on the 6-hydroxyl group in their reaction conditions using *Streptomyces* sp. alkaline protease in DMF [241]. They obtained a vinyl polymer containing trehalose in side chains (*M*_w_, 22000) after the polymerization of the 6-*O*-vinyladipoyl trehalose using azobis(2,2'-diamidino-propane)hydrochloride as a radical initiator in water (Figure 14). Miura *et al*. also reported the similar chemoenzymatic synthesis of vinyl polymers containing trehalose in side chains as a glycoconjugate polymer [242]. In addition, they prepared a polymerizable ester compound of α-d-galactopyranosyl α-d-glucopyranoside (galactose-type trehalose) with vinyl sebacate as a mimic of globosyl Gb2 ceramides. The galactose-type trehalose conjugate showed activity against Shiga toxin-1. Shiga toxins are bi-globular (AB_n_) proteins generated by *Shigella dysenteriae* and enterohemorrhagic *Escherichia coli* that inhibit protein synthesis by cleaving nucleobases from RNA [243, 244, 245].

#### Acetalization with dialdehydes

Our research on trehalose-based linear polymers started from the objective of obtaining saccharide-based linear polymers with minimum chemical reactions and without using enzymes. We focused on the highly regioselective reaction of hexose 4,6-hydroxyl groups with benzaldehyde [246]. Because trehalose has two symmetrical glucose units and is relatively stable in acids, this best fulfills the requirements to generate linear polymers from disaccharides. Acetalization reactions of 4,6-hydroxyl groups can be carried out under acidic conditions with aldehydes or their dimethyl acetals, or under basic conditions by reaction with α,α-dibromotoluene.

**Figure 15:**
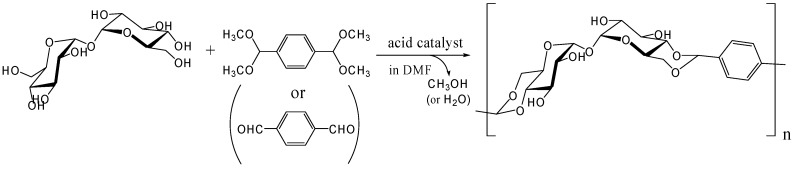
Polyacetal synthesis by the acetalization reaction of trehalose with terephthalaldehyde or its bis(dimethylacetal) form.

Our research for polymerizing trehalose in one-step was started in 2000 by reacting it with terephthalaldehyde or its bis(dimehtylacetal) form (Figure 15). The reaction conditions reported in the literature involving the synthesis of trehalose dibenzylidene acetal were modified from existing reports [247, 248] and the synthesis of the trehalose-based linear polymer was reported in 2002 and 2004, respectively [249, 250]. In these reports, *p*-toluenesulfonic acid was used as acid catalyst and a trehalose-based polyacetal *M*_w_ ~ 8,500, polydispersity 2.2 was synthesized. The similar strategy with methyl mannopyranoside was conducted around the same time by Maslinska-Solich and reported in 2001 [251]. 

**Figure 16:**
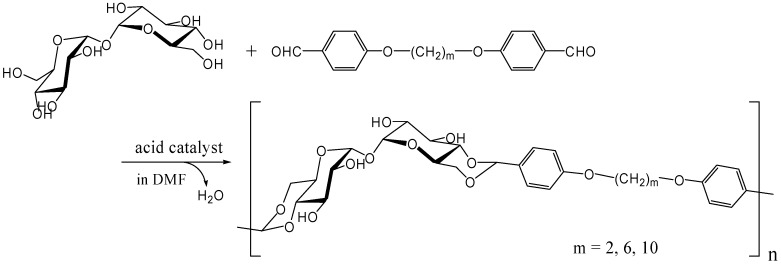
Polyacetal synthesis by the acetalization reaction of trehalose with dialdehyde compounds having two benzaldehyde units on the ends of alkylene.

The polyacetal could be synthesized directly from trehalose and terephthalaldehyde after improving the acetalization method in which water generated in the reaction was efficiently removed azeotropically with toluene using Dean-Stark distilling trap. Further study revealed that the polyacetal with a *M*_w_ up to 8,300 was synthesized by this method in the presence of Amberlyst 15 dry, a solid acidic catalyst, which gave best results among several homogeneous and heterogeneous catalysts (unpublished). As expected the polyacetal showed no glass transition temperature (*T*_g_) up to the decomposition temperature (325˚C), due to its rigid main chain. Subsequently, we synthesized dialdehyde compounds having two benzaldehyde units on ends of alkylene tethers via ether linkages to introduce flexible units in the polyacetal (Figure 16). Alkylene units used in this study were ethylene, hexylene, and decylene. The *M*_w_ of the acetalization products from trehalose and the dialdehydes were lower than 4000, though *T*_g_s were observed for the products containing alkylene units [252]. 

#### Hydrosilylation

**Figure 17:**
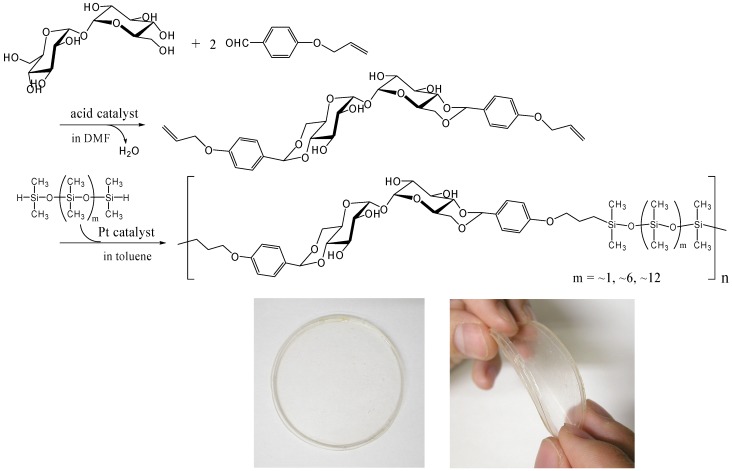
Synthesis of thermoplastic and flexible polymers containing trehalose and dimethylsiloxane oligomer units by hydrosilylation reaction (reproduced from the reference [253] with the permission of the Society of Polymer Science, Japan).

Thermoplastic properties are very important for industrial polymer synthesis, processing and recycling. We prepared a thermoplastic trehalose-based polymer by incorporating dimethylsiloxane oligomers. A trehalose-based diallyl compound synthesized by the acetalization of α,α-d-trehalose with 4-allyloxybenzaldehyde underwent hydrosilylation with SiH-terminated siloxanes in the presence of Karstedt’s catalyst (a platinum 1,3-divinyltetramethyldisiloxane complex) to provide alternating copolymers that contained trehalose units and dimethylsiloxane oligomer units (Figure 17) [253]. We used three different telechelic SiH-terminated siloxanes: 1,1,3,3,5,5-hexamethyltrisiloxane (the molecular weight, 208), and dimethylsiloxane oligomers with the molecular weight of ca. 600 and ca. 1,000. The *M*_w_ of the copolymers was up to 50,000 and all the polymers showed two *T*_g_s, suggesting microphase separation. A flexible transparent film was obtained by casting from the acetone solution of the copolymers synthesized from the trehalose-based diallyl compound and the dimethylsiloxane oligomer with the molecular weight of ca. 1,000 (Figure 17). The materials are intriguing as a biomaterial due to the biocompatibility of trehalose, and cell affinity and proliferation experiments on the material is under investigation.

#### Diels-Alder polymerization

**Figure 18:**
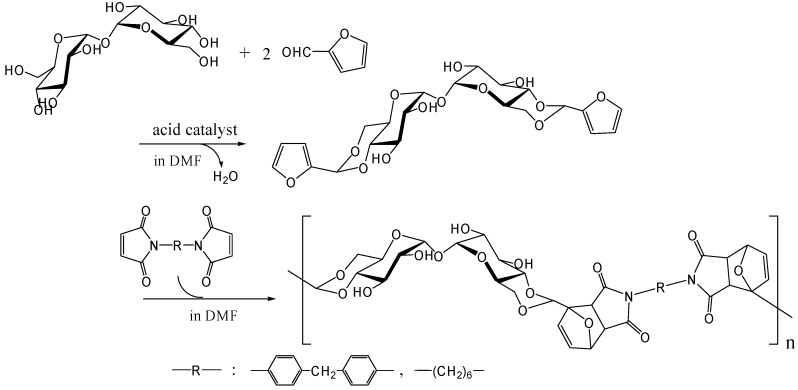
Synthesis of trehalose-based polymer by the Diels-Alder polymerization.

The polymers that allow facile recycling are also fascinating, even if the polymer does not show thermoplastic properties up to the decomposition temperature. The Diels-Alder reaction is a well-known [4+2] thermally reversible cycloaddition reaction of dienophile with diene. Polymers synthesized by Diels-Alder polymerizations thermally revert easily to monomers upon heating by the retro-Diels-Alder reaction [254]. We synthesized difurfurylidene trehalose (DFTreh) by the acetalization reaction of trehalose with furfural which is prepared from pentoses as renewable resources. We found this acetalization reaction to occur regiospecifically to give 4,6,4',6'-*O*-difurfurylidene trehalose (DFTreh). The Diels-Alder polymerization of DFTreh with bismaleimido-diphenylmethane (BMIDP) or bismaleimidohexane (BMIH) was achieved at 40 to 70˚C for 24 to 48 h [255] (Figure 18); and the reaction at 70˚C for 48 h with BMIDP produced the highest *M*_w_ of 15,800 (polydispersity of 1.5). When the reaction temperature was > 90˚C, the molecular weight was reduced because polymerization competed with the retro-Diels-Alder reaction. Polymer products from DFTreh and BMIH or BMIDP were almost completely decomposed into corresponding monomers at 140˚C as revealed by GPC analysis. We are now synthesizing alternating copolymers containing trehalose units and dimethylsiloxane oligomer units by the Diels-Alder polymerization [256].

#### Azide-alkyne Huisgen cycloaddition (click reaction)

**Figure 19:**
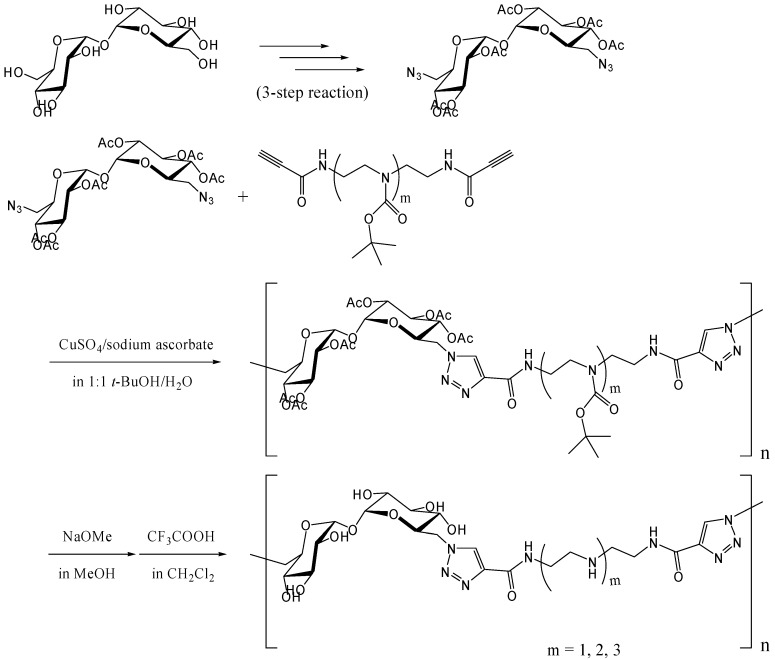
Synthesis of trehalose-based polymers containing amide-triazole groups by the click polymerization.

Reineke and coworkers synthesized trehalose-based linear polymers by the Huisgen [3+2] cycloaddition, commonly known as the "click reaction" [257, 258]. The reaction proceeds smoothly at the room temperature with copper (I) ions as catalysts [259, 260] and has many applications in organic chemistry and molecular biology, due to the mild conditions involved [261]. The polymers were designed to bind DNA for delivery of plasmid vectors into the cell. 6,6'-Diazido-6,6'-dideoxy-trehalose was synthesized, and following acetylation of remnant hydroxyl groups, it was reacted with dialkyne-oligoamine compounds to yield polymers with the *M*_w_ of up to 40,000 (a polydispersity of 1.2) (Figure 19). The polymers associated with plasmid DNA and formed polyplex with a diameter of several hundred nanometers. In the cellular uptake tests using FITC-labeled plasmid DNA and HeLa cells, the polyplex was revealed to enhance the transfection efficiency of plasmid DNA. Luciferase reporter gene was also transfected into HeLa cells using the polyplex, and the observed gene expression (in relative light unit) increased steadily as the N/P ratio (the ratio of amines (N) in the polymer/phosphates (P) in DNA) increased up to 10. The cytotoxicity assay of the polyplex revealed that the trehalose-based polymers maintained much higher cell viability at N/P = 7 than a commercially available transfection reagent based on polyethyleneimine.

#### Other methods

**Figure 20:**
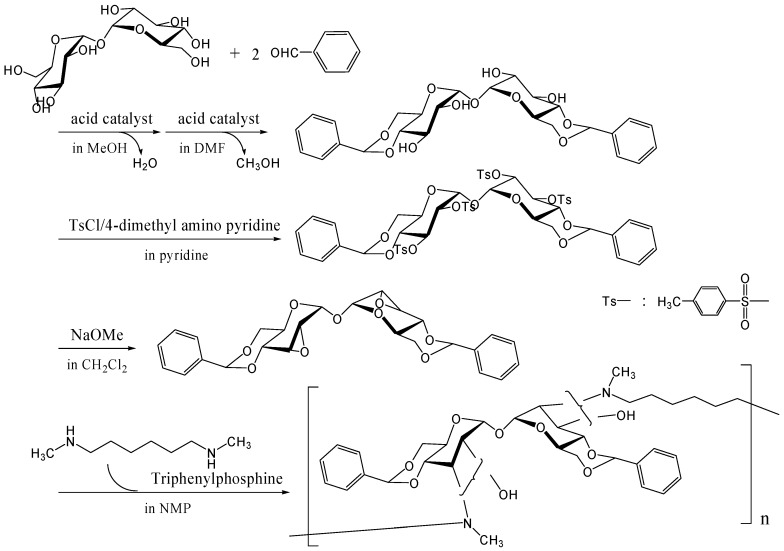
Polymerization of the trehalose-based diepoxide compound with an aliphatic diamine.

To synthesize trehalose-based cationic polymers, we synthesized a trehalose-based diepoxide compound, 2,3-anhydro-4,6-*O*-benzylidene-α-d-allopyranosyl-2,3-anhydro-4,6-*O*-benzylidene-α-d-allopyranoside, which was reacted with *N,N'*-dimethyl-1,6-diaminohexane, an aliphatic diamine, via ring opening reaction of epoxide groups (Figure 20) [262]. The trehalose-based diepoxide compound was synthesized in three-step reaction following literature protocols [248]. The reactivity of this diepoxide was not high due to the steric hindrance of epoxide groups on pyranose rings, and higher reaction temperature (200˚C) was necessary to obtain a polymer *M*_w_ of 6,500 (a polydispersity of 2.6). When 1,6-diaminohexane was reacted with the diepoxide compound, neither a gel nor a precipitate was seen in the reaction solution, suggesting that two epoxide groups hardly reacted with an identical amino group. The linear polymer can be easily converted into a cationic polymer, and the binding ability to DNA is under investigation. 

## Conclusions

The intellect of scientists working with trehalose is fed by others contributing to the vast literature on this unique disaccharide. While preparing this review we focused on delegating the essential differences between trehalose and other sugars with respect to new materials design and preparation, and found that there are astounding differences that need elucidation to expand state of the art knowledge.

We regret that all the papers discussing the glassy states induced by trehalose could not be covered in this review, and provided an in-depth discussions on the four modes of protein stabilization. The emerging issues on trehalose is increasingly spread more and more widely over a variety of fields in physical and computational chemistries, bioinformatics, biomedical sciences, tissue engineering, and material sciences. When we started the synthesis of a trehalose-based polymer for the first time, we selected it as a monomer simply for the reason that its chemical structure is suitable for our interest in polymerization and that the sugar can be easily produced from renewable resources. We find ourselves in awe of its potential in various fields of chemical biology and will start designing trehalose-based polymers on the basis of keeping its physiological properties. New paradigms will emerge, if novel trehalose-based oligomer and polymer systems that play preservation roles on proteins, membranes, cells, and organs are prepared. We are grateful to have this opportunity to make a contribution.
